# When Natural Behavior Engages Working Memory

**DOI:** 10.1016/j.cub.2020.11.013

**Published:** 2021-02-22

**Authors:** Dejan Draschkow, Melvin Kallmayer, Anna C. Nobre

**Affiliations:** 1 Oxford Centre for Human Brain Activity, Wellcome Centre for Integrative Neuroimaging, Department of Psychiatry, University of Oxford, Oxford OX3 7JX, UK; 2 Department of Experimental Psychology, University of Oxford, Oxford OX1 3UD, UK; 3 Department of Psychology, Goethe University, 60323 Frankfurt, Germany

## Abstract

Working memory (WM) enables temporary storage and manipulation of information,^1^ supporting tasks that require bridging between perception and subsequent behavior. Its properties, such as its capacity, have been thoroughly investigated in highly controlled laboratory tasks.^1^, ^2^, ^3^, ^4^, ^5^, ^6^, ^7^, ^8^ Much less is known about the utilization and properties of WM in natural behavior,^9^, ^10^, ^11^ when reliance on WM emerges as a natural consequence of interactions with the environment. We measured the trade-off between reliance on WM and gathering information externally during immersive behavior in an adapted object-copying task.^12^ By manipulating the locomotive demands required for task completion, we could investigate whether and how WM utilization changed as gathering information from the environment became more effortful. Reliance on WM was lower than WM capacity measures in typical laboratory tasks. A clear trade-off also occurred. As sampling information from the environment required increasing locomotion and time investment, participants relied more on their WM representations. This reliance on WM increased in a shallow and linear fashion and was associated with longer encoding durations. Participants’ avoidance of WM usage showcases a fundamental dependence on external information during ecological behavior, even if the potentially storable information is well within the capacity of the cognitive system. These foundational findings highlight the importance of using immersive tasks to understand how cognitive processes unfold within natural behavior. Our novel VR approach effectively combines the ecological validity, experimental rigor, and sensitive measures required to investigate the interplay between memory and perception in immersive behavior.

**Video Abstract:**

## Highlights

• Gaze provides a measure of working-memory (WM) usage during natural behavior
• Natural reliance on WM is low even when searching for objects externally is effortful
• WM utilization increases linearly as searching for objects requires more locomotion
• The trade-off between using WM versus external sampling affects performance

## Teaser

Draschkow et al. demonstrate that reliance on working memory is low during natural behavior, even when “holding information in mind to guide future behavior” is the very essence of the task. As searching for objects externally increases in locomotive effort, WM usage increases in a shallow and linear fashion. This trade-off affects performance.

## Results and Discussion

**Figure 1:**
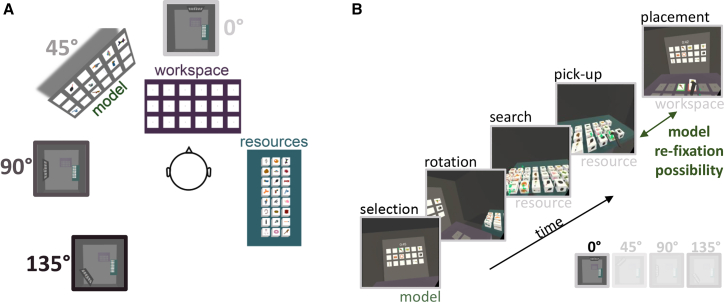
Object-Copying Task in Virtual Reality (A) Twenty-four participants copied 8 “model” arrangements of objects, here shown at 45°, by selecting and picking up objects from the “resource” section and placing them into a “workspace” area (see Video S1). The model’s location varied between conditions (1 run = 14 displays), either 0°, 45°, 90°, or 135° from the workspace. (B) Participants could only pick up and carry one object at a time, which imposed a sequential order into the task (for further details, see STAR Methods and Figure S1). Participants would (1) look at the model area in order to encode the to-be-copied object(s), (2) move to the resources in order to (3) search and select the object, (4) pick it up, and finally (5) place it in the corresponding location in the workspace. See also Figure S1 and Video S1.

In our temporally extended object-copying task (Figure 1A), participants (n = 24) copied a model display by selecting realistic objects from a resource pool and placing them into a workspace (Video S1). The immersive nature of virtual reality enabled us to disentangle different sub-parts of the completed task (Figure 1B). Critically, we varied the model’s location between conditions (0°, 45°, 90°, or 135° in relation to the workspace), thus manipulating the “locomotive effort” required between encoding objects in the model and placing the objects in the workspace (Figures 1A and S1)—a composite variable that combines the effort and time to complete the task. Other than the varying locomotive demands, the task structure remained the same across conditions.

Video S1. Temporally Extended Object-Copying Task in Virtual Reality, Related to Figure 1 and STAR Methods

### Gaze Provides a Measure of Working-Memory Usage during Natural Behavior

**Figure 2:**
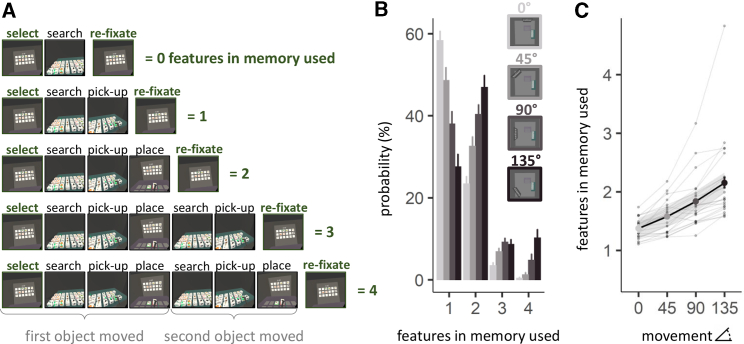
Utilization of WM Representations (A) Measuring gaze in virtual reality enabled us to develop an implicit metric of working-memory utilization (see Video S2). Because participants needed to sample identity and location information of the to-be-copied object from the model, we could count the number of WM features used in the task between re-fixations of the model. For example, if participants fixated the model *before* placing the object, they only used 1 feature, that is the identity feature of that object. If they fixated the model *after* placing the object, we counted 2 features used (both identity *and* location information were utilized). (B) Probability of using an increasing number of WM features as a function of the experimental condition (i.e., locomotive effort)—see Figure S2A for results of more than 4 features in WM. Error bars depict standard error of the mean. (C) The shape of the relationship between amount of locomotion and number of WM features utilized was linear. For a data-driven visualization of the relationship, the line in the plot was fit using a nonparametric LOESS smoothing function (thick gray line) with shaded areas representing the 95% confidence intervals. Solid dots indicate group averages and transparent lines, and dots depict the averages of the twenty-four participants. See also Figure S2 and Video S2.

Placing each object correctly required two features (i.e., types of information) in memory: its identity (1 feature) and location (1 feature). Gaze measures provided an implicit proxy of working memory (WM) utilization (see more in Figure 2A and Video S2). For example, participants could look at the model before placing the object, indicating they retained only the identity of the object, but not its location (1 feature). During another example, participants could place the object and select and place an additional object from the resource without having looked back at the model, indicating they retained the identity and locations of two objects (4 features).

Video S2. Gaze Provides a Measure of Working-Memory Usage during Natural Behavior, Related to Figure 2 and STAR Methods

Surprisingly, in the lowest locomotion condition (0°), participants relied on only one feature in memory at a time in the majority (60%) of cases. They correctly picked up an object from the resource pool (maintaining identity) but looked again at the model before placing it (sampling the location information). This result demonstrates that WM usage is lower than WM capacity estimates (∼4 items) in typical laboratory tasks.^13^, ^14^, ^15^

### Natural Reliance on WM Is Low Even When Searching for Objects Externally Is Effortful

The reliance on WM was surprisingly low, even in the most effortful case (135°). Most often, participants used two features in memory (close to 50%). Compared to the least effortful (0°) condition, the probability of using one feature dropped from 60% to less than 30% of the cases (Figure 2B; for details, see the Quantification and Statistical Analysis). Using four features also increased from ∼1% in the 0° condition to 10% in the 135° condition. Nevertheless, WM was far from loaded to the capacity derived in laboratory tasks; instead, the results demonstrated a fundamental dependence on external information during ecological behavior.

### WM Utilization Increases Linearly as Searching for Objects Requires More Locomotion

In order to describe the shape of the relationship between locomotive effort and WM utilization, we computed the average number of WM features used as a function of the different locomotion conditions (Figure 2C). Generalized linear mixed-model comparisons revealed a clear linear relationship between locomotion and the number of features used, suggesting a shift from external sampling to relying on WM (for details, see Quantification and Statistical Analysis). On average, this was a shallow change, with memory load increasing from approximately one feature in the 0° condition to two features in the 135° movement trials (Figure 2C).

### The Trade-Off between Using WM versus External Sampling Affects Performance

**Figure 3:**
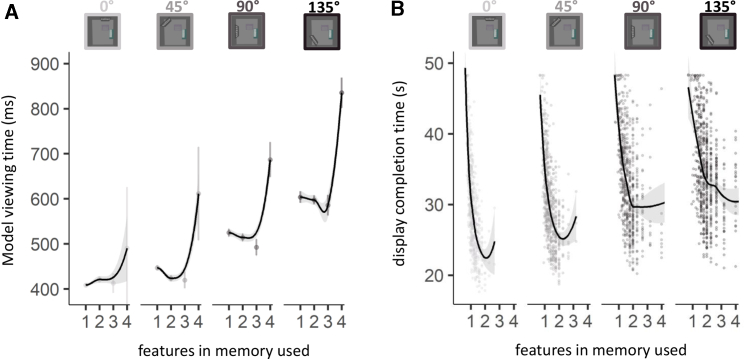
Trade-Offs between Sampling Information from WM versus from the Environment (A) Average viewing time during the initial viewing of the to-be-copied objects in the model area, as a proxy for encoding duration. Viewing times were longer for trials in which more WM features were used (positive slopes for all conditions). Conversely, viewing times also predicted the number of features used (see Figure S2B). Error bars represent 95% confidence intervals. (B) Display completion times (each point is one display completed by one participant) as a function of the average number of features used in the given display, reflecting the performance outcome of trading off using WM and external information. Greater reliance on WM reduced completion times (significant negative slopes for all conditions), though the quadratic relationship demonstrates that, with higher numbers of features in memory, performance plateaued in this task (for details, see Quantification and Statistical Analysis). For a data-driven visualization of the relationship, the lines in the plot were fit using a nonparametric LOESS smoothing function with shaded areas representing the 95% confidence intervals. The dots in (A) represent group averages, whereas the dots in (B) depict the data of individual displays. See also Figure S2.

In addition to costs related to maintaining features in WM, the time it takes to encode objects is another important factor in determining the optimal trade-off between using WM and sampling information from the environment. We found that the time participants spent viewing the model during the selection of the to-be-copied objects (Figure 3A) increased systematically as locomotive effort increased (comparing the lines between each of the facets of the figure), likely reflecting the increase in time and distance this information needed to be “carried” (for details, see Quantification and Statistical Analysis). Critically, within each locomotion condition, dwell times were longer for trials in which more WM features were used (positive linear slopes for all conditions). This relationship was quadratic (except for the line in the 0° condition [first facet], where it was linear), showing a steep increase in dwell times as participants moved from 3 to 4 features in memory (Figure 3A). The relationship between encoding duration and WM features utilized (see also Figure S2B) suggests participants *loaded-up* more information,^16^ instead of simply *using* more. This pattern highlights that, although relying on WM representations may spare locomotive effort, loading WM may bring additional costs in terms of invested time.

To provide a fuller measure of performance outcome resulting from trading off using WM versus external information, we calculated the time participants required to complete each display—that is the summed duration required to copy the 8 objects from the model area into the workspace (Figure 3B). Erroneous placements had to be corrected immediately, and fewer than 3% of copying sequences included errors (Figure S3; for details, see Quantification and Statistical Analysis). Therefore, by design, participants’ performance was evaluated by the speed of their copying behavior. For each display, we also calculated the average number of features in WM used. Figure 3B depicts this relationship as a function of the different locomotion conditions. Unsurprisingly, participants were slower to complete displays with increasing locomotion demands. However, greater reliance on WM reduced completion times (significant negative slopes for all movement conditions), though a significant quadratic relationship also revealed diminishing returns for increasing the number of features in memory, particularly when using more than ∼2 features. The observed pattern of regularly sampling information from the environment (i.e., using one feature) was therefore not the most efficient strategy, and using more features in WM increased task performance. On the other hand, encoding more features also becomes taxing in terms of encoding duration (Figure 3A), leading to diminishing returns for overall performance.

### Why Is Reliance on WM Low in Natural Behavior?

Laboratory tasks have yielded many views about capacity limits in WM relating to whether resources are continuous^6^^,^^17^^,^^18^ or discrete^7^^,^^14^^,^^19^ and whether capacity is fixed at all.^20^^,^^21^ These views are moderated by the questions of whether relevant units are integrated objects or features^7^^,^^22^^,^^23^ and whether spatial position is necessarily encoded along with specific features.^24^, ^25^, ^26^ By any of these measures, the estimates of capacity in lab tasks exceed the estimates we have obtained for the average WM load in the natural task presented here. We show that, in natural, immersive behavior, WM is costly,^27^^,^^28^ and its usage emerges in a shallow, linear fashion as locomotive effort increases.

Our findings can be reconciled with typical lab estimates in several ways. In classical WM research, objects are briefly “flashed” on a computer screen, whereas in natural behavior, we usually can decide between looking back to the objects of interest and retaining them in memory. Such sensory-motor decisions have been investigated within the framework of statistical decision theory,^29^, ^30^, ^31^ which emphasizes the importance of considering costs and benefits—mediated by the underlying neural reward circuitry^32^—in the choice of actions. Here, memory use is weighted against locomotive effort, and depending on the reliability of the representation, the actor would rely on the information in mind or update it.^33^ Looking back can be rather “cheap,” if only a few saccades are required,^34^, ^35^, ^36^ but becomes more costly in an ecological context as we have to move the head, arms, and body.^11^^,^^37^^,^^38^ Further, constrained screen-based tasks often require participants to remain still and hold gaze on a single spot. However, eye movements can disrupt visuospatial WM representations.^39^ Self-movement as well as the related computation of changing object coordinates can also reduce capacity estimates of WM^40^ and sustained attention.^41^ Additionally, programming movements in the environment might require the maintenance of relevant locations in the experimental environment, which would further reduce the WM capacity available for objects. Finally, although the overall capacity of WM is likely higher then suggested here, only some of these objects might be prioritized within WM,^42^, ^43^, ^44^ and information out of internal focus may be susceptible to interference when interacting with distractor objects in the resource area. In sum, resource allocation in natural tasks can be vastly different from utilizing resources in laboratory settings, highlighting the need to understand how cognitive processes unfold within natural behavior.

### Using versus Not-Using Memories from Different Timescales

Although classical laboratory tasks measure the upper bound of what is possible (i.e., benchmarking), here, we aim to describe how the usage of this cognitive resource emerges during natural behavior as a function of locomotive effort—providing a measure of how WM capacity is used^12^ rather than its maximum potential. Using versus not-using memory has been investigated more thoroughly with respect to long-term memory (LTM) guidance of visual search.^45^, ^46^, ^47^, ^48^, ^49^, ^50^, ^51^, ^52^, ^53^ This literature provides evidence that, when searching for a target requires only a few fixations, LTM use can be low, whereas effortful (requiring more time and distance) searches in immersive environments recruit a more substantial usage of long-term representations.

Real-world cognition, however, is not restricted to perception, WM, or LTM operating in isolation. Instead, the content from these different timescales is integrated to serve adaptive and purposeful behavior.^54^ Understanding how information collected over these different timescales work together or compete to guide successful adaptive behavior is an exciting prospect and remains to be addressed in future research. The task we present here offers the opportunity to address these questions while taking into account environmental constraints and energetic costs, thus recognizing—instead of ignoring—the functional and ecological aspects of cognition.^33^^,^^55^

### When Natural Behavior Engages WM

Our novel VR task provides a useful naturalistic setting in which the observer is required to (1) actively *maintain* visuospatial representations in WM, (2) *protect* them from the interference of visual translations (rotating through the environment) and interference from similar objects (distractor objects in the resource area), and (3) *manipulate* the contents in memory in order to update the computation of changing object coordinates. We further use (4) realistic novel objects,^56^ rather than intrinsically confusable and hard-to-remember colored blocks,^12^^,^^57^ in order to adhere more closely to naturalistic constraints. These points constitute a combination of the challenges that have been a hallmark of active WM usage and enable an ecological investigation of the WM properties necessary for bridging between perception and subsequent behavior.

Our novel VR approach provides an ideal starting point for investigations into the factors and mechanisms that support memory usage in naturalistic settings. For example, manipulating the stimuli and their arrangements will inform the role of intrinsic memorability^58^, ^59^, ^60^ when using memory. Further, increasing the granularity of the eye-movement recordings and changing the task relevance of location and identity object features^7^^,^^22^, ^23^, ^24^^,^^26^ will show whether using some features is costlier than others. Finally, directly comparing WM usage estimates from our task with capacity and precision estimates from standard laboratory tasks will elucidate the relationship between the upper bounds of capacity,^5^ controlling access to WM,^61^ and the amount of memory actually used. Such investigations would inform how memory use is related to individual differences in cognitive capacities in a broader sense, specifically how natural memory use relates to IQ and academic attainment^62^ as well as mental workload^63^ in more applied settings.

### Conclusions

Our estimates show remarkably low levels of WM utilization in immersive behavior, even when “holding information in mind to guide future behavior” is the very essence of the task. We found reliance on ∼1 feature in WM when locomotive demands were at their lowest, which increased to an average of ∼2 features in memory at the highest locomotive demands we tested. Encoding more features bore a cost, as suggested by viewing times of the information to be maintained. Although this cost made the individual copying sequences last longer, it also enabled more information to be copied in each sequence, which reduced the overall completion times of the to-be-copied displays. This increase in efficiency, however, plateaued at ∼2 to 3 features in memory, demonstrating the importance of balancing the reliance on WM with gathering information externally during immersive behavior. Our novel VR approach effectively combines the ecological validity, experimental rigor, and sensitive measures required to investigate the interplay between memory and perception in natural behavior and opens the doors to many interesting future investigations.

## STAR★Methods

### Key Resources Table

**Table:** 

REAGENT or RESOURCE	SOURCE	IDENTIFIER
**Deposited Data**		

Post-processed data and R code	https://osf.io/sbzt6/	https://doi.org/10.17605/OSF.IO/SBZT6

**Experimental Models: Organisms/Strains**		

Twenty-four healthy human volunteers participated in the study (mean age = 26.2, range = 18-36, 17 female, all right-handed)	N/A	N/A

**Software and Algorithms**		

Lmer(), Glmer()	^64^	https://doi.org/10.18637/jss.v067.i01
Wrapper for Lmer(), Glmer()	^65^	https://dx.doi.org/10.18637/jss.v082.i13
R	^66^	http://www.r-project.org
RStudio	^67^	https://rstudio.com/
ggplot2()	^68^	https://ggplot2.tidyverse.org/
Box-Cox Trasformation	^69^	https://www.jstor.org/stable/2984418
pairedSamplesTTest()	^70^	https://learningstatisticswithr.com/book/

**Other**		

HTC Vive Tobii Pro VR	Tobii	https://www.tobiipro.com/de/produkte/vr-integration/
The Novel Object and Unusual Name (NOUN) Database	^56^	https://doi.org/10.3758/s13428-015-0647-3

### Resource Availability

#### Lead Contact

Further information and requests for resources should be directed to and will be fulfilled by the Lead Contact, Dejan Draschkow (dejan.draschkow@psych.ox.ac.uk).

#### Materials Availability

No materials are available for this study.

#### Data and Code Availability

The data and code generated during this study are available at Open Science Framework: https://osf.io/sbzt6/

### Experimental Model and Subject Details

Twenty-four healthy human volunteers participated in the study (mean age = 26.2, range = 18-36, 17 female, all right-handed). All participants had normal or corrected-to-normal (6 participants used lenses) vision and reported no history of neurological or psychiatric disorders. Participants received financial compensation (£10/h) and provided informed consent prior to participating in the experiment. Protocols were approved by the local ethics committee (Central University Research Ethics Committee #R64089/RE001).

### Method Details

#### Apparatus and Virtual Environment

Participants wore an HTC Vive Tobii Pro VR integration with a built-in binocular eye tracker with an accuracy of approximately 0.5° visual angle. We tracked gaze position in 3D space at a sampling rate of 90 Hz. Gaze position in 3D space was obtained by intersecting the gaze vector with objects in the environment. The head-mounted display (HMD) consisted of two 1080 × 1200 pixel resolution OLED screens (refresh rate = 90 Hz, field-of-view = 100° horizontally × 110° vertically). Locations of the headset and the hand-held controller were tracked with sub-millimeter precision using two Lighthouse base stations that emitted infrared pulses, which were detected by 37 infrared sensors in the HMD and 24 in the controller. Tracking was optimized by an accelerometer and a gyroscope embedded in the HMD. A trigger button (operated with the index finger) and a grip button (operated with the thumb) on the wireless controller were used for interacting with the experimental program. By intersecting the controller with a virtual object and holding down the trigger button, the participants could pick up objects – releasing the trigger button released the object from the virtual grip.

The virtual environment was presented and rendered with Unity on a high-performance PC running Windows 10 (Figures 1A and S1). The environment consisted of a 450 × 450 cm room with a ceiling height of 240 cm. Participants were situated in the center of the room, with the Model (120 × 60 cm), Workspace (100 × 50 cm) and Resources (120 × 75 cm) surrounding them.

The objects participants handled were cubes, with each side of the cube spanning 10x10 cm. The placeholders in the Model area and each side of the cube of the objects in the Resource area were overlaid with images from the Novel Object and Unusual Name Database.^56^ These stimuli hold the advantage of being naturalistic, while at the same time unfamiliar and difficult to verbalize. Out of a stimulus pool of 60 objects, 8 objects were randomly selected for the Model area and 16 additional objects were drawn for the Resources, for each display. The location assignment of all objects was pseudo-randomized, so that a specific display arrangement never repeated across runs for a participant.

#### Procedure and tasks

Upon arrival, participants were informed that they would perform an object-copying task in which they would have to: (1) find the objects depicted in the Model within the Resources; (2) pick up these objects and move them into the Workspace to copy the arrangement in the Model; and (3) complete each display as quickly as possible (with a timeout of 45 s per display).

The Model contained the configuration of objects to be copied; the Resource contained the objects to be used; and the Workspace was the area in which the copied arrangement was assembled (Figures 1A and S1; Video S1). Participants could only pick up and carry one object at a time with their controller, which imposed a sequential order into the task (Figure 1B). The picked-up object needed to be placed in the appropriate location of the Workspace. Once the participant successfully placed the object, the location was highlighted with green contours (Video S1). Red contours would signal to the participant if the wrong location was chosen (Figure S3). The objects in the Resource would be rendered invisible as long as an object was placed incorrectly in the Workspace, thus making it impossible for the participant to continue until the object was either placed correctly, or removed from the Workspace (e.g., an incorrect object was moved to begin with). By design, participants performance was evaluated by the speed of their copying behavior, because erroneous placements had to be corrected immediately and fewer than 3% of copying sequences contained errors (Figure S3). On average, 1.8% of the sequences contained an identity error (picking up an object which was not contained in the Model) and 2.9% contained a location error (placing an object on the incorrect field). In case objects fell on the ground during copying, they would automatically re-spawn in the Resource.

Critically, we varied the Model’s location between runs (0°, 45°, 90°, or 135° in relation to the Workspace), thus manipulating the ‘locomotive effort’ required between encoding objects in the Model and placing the objects in the Workspace (Figures 1A and S1)) – a composite variable that combines the effort and time to complete the task. Other than the varying locomotive demands, the task structure remained the same across conditions.

A short practice session familiarised participants with the HMD, the wireless controller, the testing procedure, and the lab space. The practice session was identical to the actual task and consisted of copying all objects in 3 displays. Practise continued until all open questions were resolved.

Participants completed 8 runs of trials split between two sessions. Within each run they reproduced 14 displays, each display containing 8 to be copied objects. The experimental manipulation (0°, 45°, 90°, or 135°) was varied run-wise, so that every run consisted of trials from a single condition. A mandatory break (approximately 5 minutes) was administered after completing the first four runs (session 1). During the break participants removed the headset and could rest. In the second session, participants completed four more runs. Thus, each participant completed 28 displays (copied 224 objects) per condition (0°, 45°, 90°, and 135°). The order of the conditions was randomized across sessions and participants. The full experiment lasted approximately 90 minutes.

### Quantification and Statistical Analysis

#### Data recording and pre-processing

Data from 24 displays across participants were removed due to missing values. All remaining data from 2664 displays were included in the analysis.

Frame-by-frame data were written to a csv file during recording. For the purpose of the data analysis, we segmented our measures of interest into *sequence*. A sequence always started by detecting a gaze sample on the Model and ended with a gaze sample on the Model. Critically, to qualify as a sequence, the participants must have looked at either the Model or Resources, between two views of the Model. For example, a sequence could consist of an initial gaze to the Model, followed by a gaze to the Resource, and finally conclude with another gaze back to the Model. This final gaze sample concludes this sequence and initiates the next sequence.

#### Number of features in memory

We quantified the number of features in memory during each sequence, according to the actions performed (Figure 2A; Video S2). Specifically, we considered how many object features (identity, location) were acted upon, before observers’ gaze returned to the Model for additional encoding. If, for example, the only action that was performed during a sequence was picking up an object from the Resource, this was counted as a 1-feature sequence. That is, the participant looked at the Model *before* placing the object. In a 2-features sequence, for example, an observer not only picked up the object, but also placed it in the Workspace before their gaze returned to the Model area. If a second object was picked up before looking back to the Model, this would constitute that 3 features in memory were used, and if the second object was also placed then this would be categorized as a 4-feature sequence, etc. For each display, participants had to place 8 objects. The overall number of features per display added up to 16 features since each object contained 2 features: 1 identity + 1 location feature (Figure S2A).

As participants’ behavior in this unconstrained task is not perfectly characterized by this categorization, we also counted cases in which 0 features were used. This captures cases in which no object was picked up during a sequence (Figure S2A). While this metric captures occurrences in which participants genuinely looked at the Model, turned to the Resource, and realized that they did not remember what they are looking for; it is likely also strongly contaminated by positional adjustments, lapses of attention, reorienting, and other unforeseen idiosyncrasies.

#### Model viewing time

Model viewing times were derived individually for each sequence. Thus, each viewing time had a corresponding features-in-memory value.

#### Display completion time

To calculate display completion time, we summed up the individual sequence completion times for each display.

#### Data analysis

The descriptions in this section are organized according to the figures in the main text and supplementary materials (see Data and Code Availability for access to data and code). Analyses were run using the *lsr* package,^70^ *lme4* package^64^ and *lmerTest*^65^ in the R statistical programming language^66^ using RStudio.^67^ The *ggplot2* package^68^ was used for data visualization and the *MASS* package^71^ for conducting the Box–Cox procedure.^69^

All mixed-effects models were fitted with the maximum likelihood criterion. After inspecting the distributions of dependent variables, their residuals, and power coefficient outputs, we transformed the values in order to approximate a normal distribution more closely – here the Box–Cox procedure suggested a log transformation for all relevant continuous variables. Predictor variables were z-transformed (scaled and centered) and where relevant higher-order orthogonal polynomials were evaluated (e.g., linear, quadratic, or cubic).

#### Statistical analysis related to Figure 2B – probability of using features in memory

Differences between means of conditions were analyzed using planned pairwise t tests. Planned comparisons revealed a significant difference between the probability values of all neighboring movement conditions, nested within each number of features in memory (see table below). Only the difference between 90° and 135° for 3-features in memory was not reliable (p = 0.5).

**Table:** 

*Row*	Measure	Comparison	t	*df*	p	Cohen’s *d*	Mean Diff	95% CI
*1*	probability	1 feature: 0° versus 45°	4.381	23	< 0.001	0.894	9.754	5.149, 14.359
*2*	probability	1 feature: 45° versus 90°	4.626	23	< 0.001	0.944	10.564	5.84, 15.289
*3*	probability	1 feature: 90° versus 135°	6.475	23	< 0.001	1.322	10.475	7.129, 13.822
*4*	probability	2 features: 0° versus 45°	−4.804	23	< 0.001	0.981	−9.153	−13.094, −5.212
*5*	probability	2 features: 45° versus 90°	−3.375	23	.003	0.689	−7.754	−12.508, −3.001
*6*	probability	2 features: 90° versus 135°	−3.665	23	.001	0.748	−6.598	−10.323, −2.874
*7*	probability	3 features: 0° versus 45°	−5.042	23	< 0.001	1.029	−3.387	−4.777, −1.998
*8*	probability	3 features: 45° versus 90°	−3.064	23	.005	0.625	−2.345	−3.928, −0.762
*9*	probability	3 features: 90° versus 135°	0.681	23	.503	0.139	0.595	−1.214, 2.405
*10*	probability	4 features: 0° versus 45°	−2.382	23	.026	0.486	−0.875	−1.634, −0.115
*11*	probability	4 features: 45° versus 90°	−3.064	23	.005	0.625	−3.547	−5.942, −1.152
*12*	probability	4 features: 90° versus 135°	−5.63	23	< 0.001	1.149	−5.432	−7.428, −3.436

Each row shows the results of a within-subject paired t-test.

#### Statistical analysis related to Figure 2C – number of features in memory

Generalized linear mixed-effects models (GLMMs) with a Poisson distribution were used to investigate how the experimental manipulation (from 0° to 135°) predicted the number of features in memory used. The random effects structure included a by-participant random intercept and a by-participant random slope for the locomotive condition. A model using a third-order polynomial (linear, quadradic and cubic) demonstrated that an increase in locomotive demands significantly predicted the number of features in memory, only when modeled with a first order polynomial (linear) fit, β = 24.254, SE = 2.393, *z* = 10.134, p < 0.001 (compared to quadratic, p = 0.493 and cubic, p = 0.818). To be exhaustive, we performed model comparisons on an array of plausible models, which are summarized in the following table.

**Table:** 

*Row*	Measure	Predictor	*df*	*AIC*	*BIC*	logLik	χ^2^	*p*
*1*	features in memory	exponential (locomotion)	5	69392	69433	−34691	—–	—–
*2*	features in memory	logarithmic (locomotion)	5	69397	69438	−34694	0	1
*3*	features in memory	linear (locomotion)	5	69358	69399	−34674	39.780	< 0.001
*4*	features in memory	quadratic (locomotion)	6	69359	69408	−34674	0.448	.503
*5*	features in memory	cubic (locomotion)	7	69361	69419	−34674	0.052	.820

Each row shows the results from a likelihood ratio procedure comparing each model with the preceding one.

Higher order polynomial fits always included the lower order polynomials.

#### Statistical analysis related to Figures 3A and S2B – Model viewing time

To analyze viewing durations during the initial viewing of the Model, we restricted our analysis to instances in which between 1 and 4 features in memory were used. We excluded sequences with viewing times below 50 ms and above 2000 ms, which excluded less than 1% of the data.

Summed initial viewing time of the Model area was added as a predictor to the best fitting GLMM modeling the number of features in memory used (section above). Viewing times significantly predicted the number of features used (Figure S2B), β = 0.014, SE = 0.006, *z* = 2.377, p = 0.017, but did not interact with locomotion, β = 0.007, SE = 0.005, *z* = 1.337, p = 0.181. The effect of locomotion remained significant after the inclusion of the new predictor, β = 0.131, SE = 0.011, *z* = 11.423, p < 0.001.

To investigate how the number of features in memory as well as locomotion influenced viewing times, we used linear mixed-effects models (Figure 3A). With respect to the random effects of the models, we started with maximal^72^ random effects structures which included by-participant random intercepts and by-participant random slopes for the effect of locomotion, the effect of features in memory and the interaction of these two. The number of features in memory predictor entered both the fixed and the random effects structure with a linear, as well as quadratic fit (Figure 3A). Full models often lead to overparameterization and convergence issues.^73^ We ran a principal component analysis (PCA) of each fitted model’s random-effects variance-covariance estimates to identify overparameterization and then removed random slopes that were both (a) not supported by the PCA and (b) did not contribute significantly to the goodness of fit as assessed by a likelihood ratio test comparing models with and without the slope in question. No model simplification was justified after simplification of the random-effects structure; thus, we report the inferential outcomes of the full model.

**Table:** 

*Row*	Measure	Predictor	t	*df*	*p*	β	SE
*1*	Viewing time	linear (features in memory)	1.600	23.55	.123	1.397	0.087
*2*	Viewing time	quadratic (features in memory)	3.328	23.21	.003	2.890	0.087
*3*	Viewing time	locomotion	11.361	22.63	< 0.001	0.145	0.013
*4*	Viewing time	linear (features in memory): locomotion	0.039	22.37	.97	0.030	0.763
*5*	Viewing time	quadratic (features in memory): locomotion	3.148	24.80	.004	2.032	0.645

Each row shows the results of a predictor variable.

The *p-value*s were calculated with the Satterthwaite’s degrees of freedom method.

To clarify the interaction (row 5) between locomotion and the number of features in memory (modeled with a quadratic fit), we calculated separate models for each locomotion condition.

**Table:** 

*Row*	Measure	Predictor	t	*df*	*p*	β	SE
*1*	Viewing time	0°: linear (features in memory)	3.179	22.31	< 0.001	1.804	0.567
*2*	Viewing time	0°: quadratic (features in memory)	−0.009	3085	.993	−0.003	0.385
*3*	Viewing time	45°: linear (features in memory)	−1.835	2946	.067	−0.757	0.413
*4*	Viewing time	45°: quadratic (features in memory)	1.069	18.32	.299	0.848	0.793
*5*	Viewing time	90°: linear (features in memory)	1.072	23.26	.295	0.834	0.779
*6*	Viewing time	90°: quadratic (features in memory)	4.436	25.88	< 0.001	2.332	0.525
*7*	Viewing time	135°: linear (features in memory)	3.479	21.95	.002	2.537	0.729
*8*	Viewing time	135°: quadratic (features in memory)	7.192	1329	< 0.001	3.159	0.439

Each row shows the results of a predictor variable.

The *p-value*s were calculated with the Satterthwaite’s degrees of freedom method.

Viewing times were longer for trials in which more WM features were used (positive slopes for all conditions).

#### Statistical analysis related to Figure 3B – display completion time

For analyzing the overall time it took participants to complete a display, we summed the individual sequence completion times for each display (Figure 3B). We also calculated the average number of features in memory used for each display, enabling us to relate these measures.

To predict display completion time, the linear mixed-effects model included the predictors locomotion and number of features in memory. The random-effects structure consisted of by-participant random intercepts and by-participant random slopes for the effect of locomotion, the effect of features in memory and the interaction of these two. The number of features in memory predictor entered both the fixed and the random-effects structure with a linear, as well as quadratic fit (Figure 3B).

**Table:** 

*Row*	Measure	Predictor	t	*df*	*p*	β	SE
*1*	Completion time	linear (features in memory)	−2.735	2.73	.079	−1.166	0.427
*2*	Completion time	quadratic (features in memory)	15.078	14.97	< 0.001	7.820	0.519
*3*	Completion time	locomotion	20.397	21.09	< 0.001	0.129	0.006
*4*	Completion time	linear (features in memory): locomotion	−6.840	6.457	< 0.001	−2.918	0.427
*5*	Completion time	quadratic (features in memory): locomotion	−10.085	3.334	.001	−3.784	0.375

Each row shows the results of a predictor variable.

The *p-value*s were calculated with the Satterthwaite’s degrees of freedom method.

To clarify the interactions (row 4 and 5) between locomotion and the number of features in memory, we calculated separate models for each locomotion condition.

**Table:** 

*Row*	Measure	Predictor	t	*df*	*p*	β	SE
*1*	Completion time	0°: linear (features in memory)	−19.90	647.97	< 0.001	−2.513	0.126
*2*	Completion time	0°: quadratic (features in memory)	8.44	634.74	< 0.001	0.907	0.107
*3*	Completion time	45°: linear (features in memory)	−16.84	666.64	< 0.001	−2.281	0.135
*4*	Completion time	45°: quadratic (features in memory)	10.13	650.40	< 0.001	1.065	0.105
*5*	Completion time	90°: linear (features in memory)	−6.824	23.29	< 0.001	−2.855	0.418
*6*	Completion time	90°: quadratic (features in memory)	4.359	119.78	< 0.001	0.841	0.193
*7*	Completion time	135°: linear (features in memory)	−6.532	4.88	.001	−1.115	0.170
*8*	Completion time	135°: quadratic (features in memory)	8.175	3.91	.001	2.346	0.287

Each row shows the results of a predictor variable.

The *p-value*s were calculated with the Satterthwaite’s degrees of freedom method.

## Supplemental Information

Document S1. Figures S1–S3

Document S2. Article plus Supplemental Information

Video Abstract
