# Supplementary material for: When Natural Behavior Engages Working Memory
Source: Curr Biol. 2021 Feb 22;31(4):869–874.e5. doi: 10.1016/j.cub.2020.11.013 (PMC7902904; doi:10.1016/j.cub.2020.11.013)
Supplement: Document S1. Figures S1–S3 [file mmc1.pdf]

Current Biology, Volume *31*

Supplemental Information

When Natural Behavior Engages Working Memory

Dejan Draschkow, Melvin Kallmayer, and Anna C. Nobre

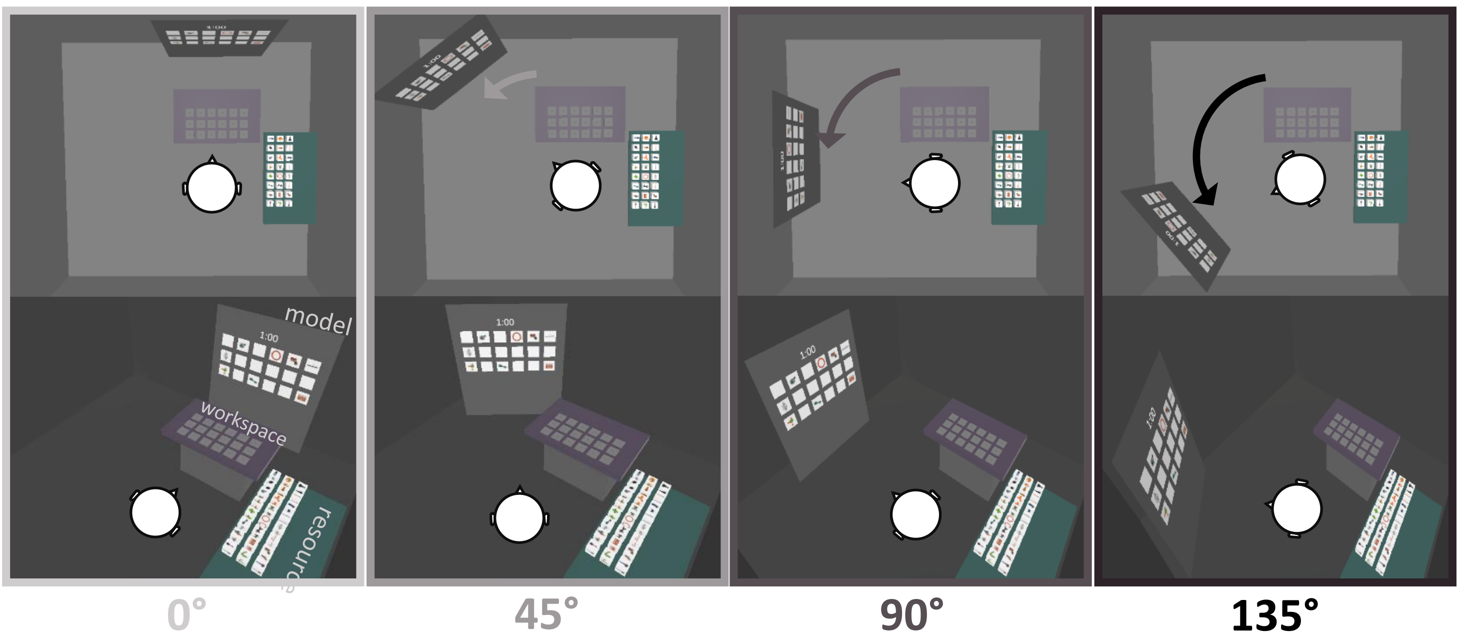

Figure S1. **The four locomotion conditions in the experiment, Related to Figure 1 and STAR Methods.** Participants completed 8 runs of trials split between two sessions. Within each run they copied 14 displays, each display containing 8 to be copied objects. The experimental manipulation (0°, 45°, 90°, or 135°) was varied run-wise, so that every run consisted of trials from a single condition. The order of the runs was randomized across sessions and participants.

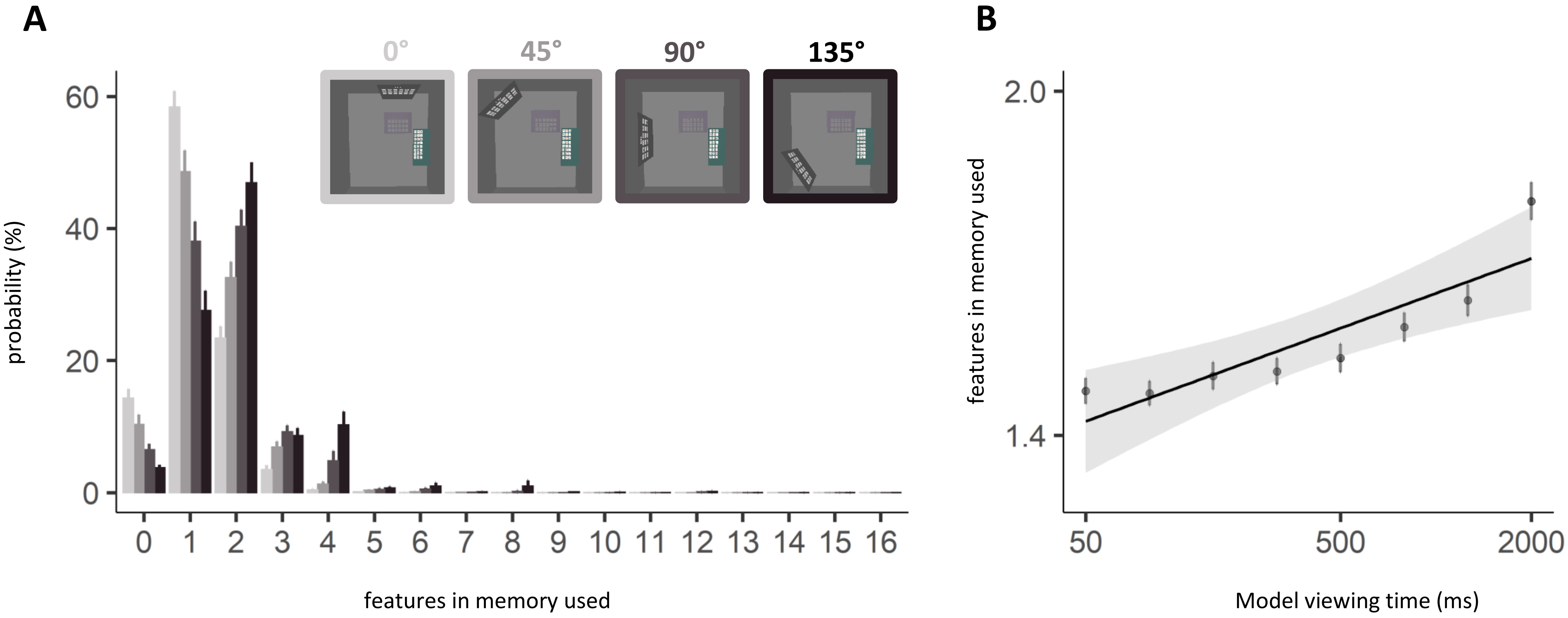

Figure S2. **Utilization of WM representations (A) and relationship between encoding duration and WM features utilized (B), Related to Figure 2, Figure 3, and STAR Methods. (A)** Probability of using an increasing number of WM features as a function of the experimental condition (locomotive effort). Error bars depict standard error of the mean. For each display, participants had to place 8 objects. The overall number of features per display added up to 16 features, because each object counted as 2 features: 1 identity + 1 location feature. In comparison to **Figure 2B** the probabilities for all 16 features in memory are depicted. There were individual cases in which participant's used more than 4 features – mostly in the 135° condition – this was a very rare occurrence. Here we also show data for 0 features in memory used. Note, that while this metric captures occurrences in which participants genuinely looked at the Model, turned to the Resource and realized that they did not remember what they are looking for, it is likely strongly contaminated by noise. That is, positional adjustments, lapses of attention, reorienting, and other unforeseen idiosyncrasies. **(B)** Predicting the number of features in WM used with the time spent looking at the model before each move sequence. Viewing times significantly predicted the number of features used. The data was binned and averaged for visualization but entered the analysis as continuous (for details see **Quantification and Statistical Analysis**). Shaded areas are representing the 95% confidence intervals of a linear fit. Error bars depict 95% confidence intervals.

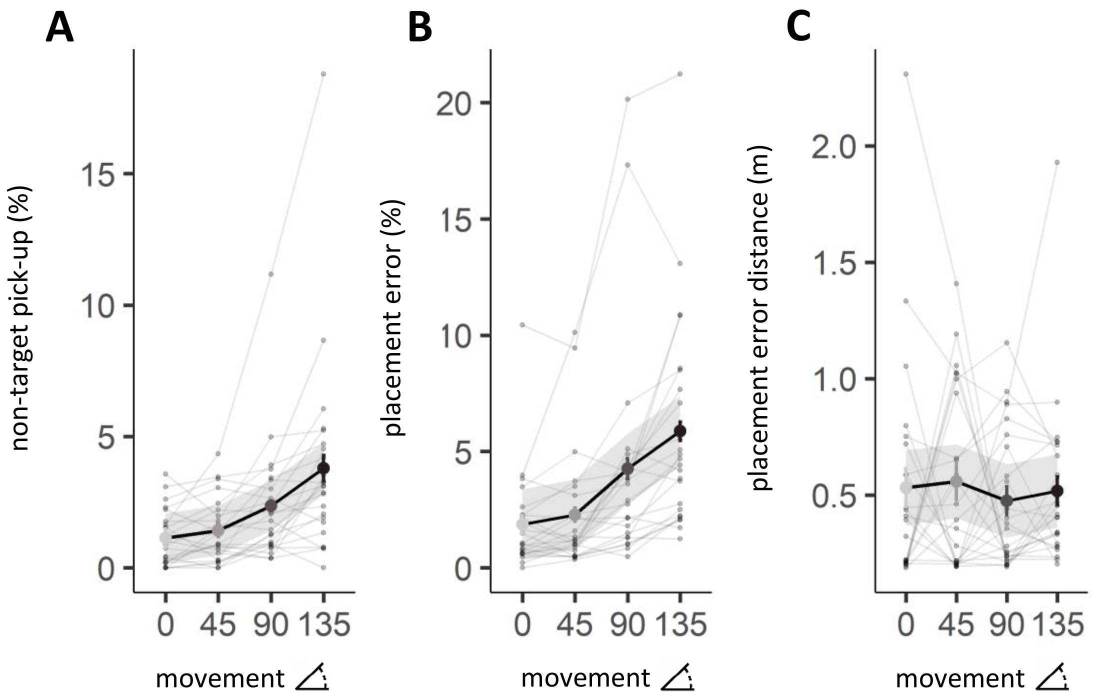

Figure S3. **Errors made during object copying, Related to STAR Methods.** All panels depict the shape of the relationship between amount of locomotion and the errors made during object copying. (**A**) On average, 1.8% of the sequences contained a non-target pick-up (picking up an object which was not contained in the Model). A GLMM revealed that locomotive effort significantly predicted the proportion of errors made, $ß = 85.325$, SE = 13.029, $z = 6.549$, $p < 0.001$. (**B**) On average, 2.9% of the sequences contained a location error (placing an object on the incorrect field). Locomotive effort significantly predicted the proportion of errors made, $ß = 84.843$, SE = 13.029, $z = 7.000$, $p < 0.001$. The optimal fit for both (**A**) and (**B**) constituted a linear relationship. (**C**) Contingent on participants actually making a placement error, we also calculated the distance of the placement from the correct location of the object. This continuous metric of location error was not predicted by locomotive effort, $t < 1$. Note, that this metric is only based on 2.9% of the sequences (when participants made a placement error) and is thus likely underpowered. The lines in the plot were fit using a nonparametric LOESS smoothing function with shaded areas representing the 95% confidence intervals. Solid dots indicate group-averages and transparent lines, and dots depict the averages of the twenty-four participants.
